# Immune checkpoint inhibitors alone or in combination with chemotherapy for treatment of advanced non-small cell lung cancer after first-line platinum-based chemotherapy: A propensity score matching analysis

**DOI:** 10.3389/fonc.2022.974227

**Published:** 2022-11-29

**Authors:** Lupeng Qiu, Shan Gao, Sicheng Du, Shengjie Sun, Yanjie Liang, Zhuoya Sun, Tao Li, Guhe Jia, Ke Li, Xiaohui Sun, Shunchang Jiao, Xiao Zhao

**Affiliations:** ^1^ Department of Oncology, The First Medical Center of Chinese PLA General Hospital, Beijing, China; ^2^ Department of Graduate Administration, Chinese People’s Liberation Army (PLA) General Hospital, Beijing, China; ^3^ School of Medicine, Nankai University, Tianjin, China; ^4^ Department of Radiotherapy, The First Medical Center of Chinese PLA General Hospital, Beijing, China

**Keywords:** non-small cell lung cancer, immune checkpoint inhibitor, chemotherapy, propensity score matching, overall survival

## Abstract

**Background:**

Immune checkpoint inhibitors (ICIs) have changed the treatment landscape of several cancer types. However, data are lacking with regard to the clinical responsiveness of ICIs in patients with advanced non-small cell lung cancer (NSCLC) after standard first-line chemotherapy. Therefore, we aimed to evaluate the clinical efficacy of ICI alone or in combination with chemotherapy for patients with advanced NSCLC after first-line platinum-based chemotherapy.

**Methods:**

We retrospectively collected patients with confirmed advanced NSCLC who underwent ICI monotherapy or ICI plus chemotherapy after first-line platinum-based chemotherapy between January 2018 and December 2020. A propensity score matching analysis was used to balance baseline characteristics between the two treatment groups. Kaplan-Meier methods and multivariable Cox regressions were used for survival analyses.

**Results:**

Among 832 eligible patients, 222 received ICI monotherapy and 610 received ICI plus chemotherapy. The median overall survival (OS) of patients who received ICI plus chemotherapy was 16.0 months compared with 13.1 months in patients who received ICI monotherapy (HR: 0.64, 95% CI: 0.49-0.85, P = 0.002). After 1:1 propensity score matching, all baseline characteristics were well-balanced between the two treatment groups. Patients who received ICI plus chemotherapy had significantly longer OS than those who received ICI monotherapy (NR vs. 13.1 months, HR: 0.50, 95% CI: 0.34-0.71, P < 0.001). Meanwhile, the median time to treatment discontinuation was 4.4 months in the ICI-chemo group and 3.5 months in the ICI-mono group (HR: 0.72, 95% CI: 0.58-0.89, P = 0.002). The multivariate analysis indicated that treatment regimen was an independent prognostic factor for OS (HR: 0.488, 95% CI: 0.337-0.707, P < 0.001). Moreover, a nomogram that integrated both treatment regimens and clinicopathological factors was created for survival prediction.

**Conclusion:**

Our study indicated that patients with advanced NSCLC who received ICI plus chemotherapy after first-line platinum-based chemotherapy tended to have longer OS than those who received ICI monotherapy. The multivariate analysis showed that treatment regimen was an independent prognostic factor for OS. Future prospective studies are needed to confirm these findings.

## Introduction

Lung cancer is the leading cause of cancer-related mortality worldwide, with non-small cell lung cancer (NSCLC) comprising 80-85% of lung cancers (1–3). Despite declines in mortality related to NSCLC in the past few years, it is still a serious threat to global health (4, 5). Currently, there are numerous treatment options for patients with NSCLC including surgery, chemotherapy, radiation therapy, targeted therapy, and immunotherapy (6–10). However, the prognosis remains poor for most patients, with a 5-year survival rate of only 22% (11). First-line treatment for advanced NSCLC patients without targetable mutations is generally based on platinum-based combination chemotherapy, with a modest median progression-free survival (PFS) of 2-4 months and overall survival (OS) of 8-10 months (12). In the past, limited effective treatment options existed for patients with progression of disease or suboptimal tumor response after first-line platinum-based chemotherapy. With the recent advent of molecular targeted therapy and immunotherapy, the five-year survival rate for patients with advanced NSCLC continues to improve (13, 14). Currently, the use of immune checkpoint inhibitor (ICI), alone or in combination with chemotherapy, has become a valid second-line treatment option for advanced NSCLC patients without targetable mutations after first-line platinum-based chemotherapy.

Immune checkpoint inhibitors, represented by programmed death 1 (PD-1) and programmed death ligand 1 (PD-L1) inhibitors, target the regulatory pathway of T cells to enhance antitumor immunity, and these agents showed durable clinical benefits and long-term remissions in some advanced NSCLC patients (15–17). The KEYNOTE-189 and KEYNOTE-407 studies showed that pembrolizumab plus chemotherapy improved OS in squamous and nonsquamous NSCLC (18, 19). Similarly, nivolumab also has promising efficacy for the treatment of patients with advanced NSCLC (20, 21). Therefore, the Food and Drug Administration (FDA) approved PD-1/PD-L1 inhibitors, such as pembrolizumab, nivolumab, and atezolizumab, for the treatment of NSCLC (**Table S1**). In addition, at least three native PD-1 inhibitors, including sintilimab, toripalimab, and camrelizumab, are also available for advanced NSCLC patients in China (22–24). ICIs have changed the treatment landscape of patients with advanced NSCLC over the last decade.

Although the results derived from clinical trials are essential in determining the efficacy and safety of different treatment regimens, patients enrolled based on restrictive selection criteria are not representative of the entire population of patients in a real-world setting (25, 26). In fact, data are lacking with regard to the clinical responsiveness of patients with advanced NSCLC who were treated with ICI monotherapy or ICI plus chemotherapy after first-line platinum-based chemotherapy, especially in the Chinese population. Therefore, we conducted a retrospective propensity score-matched (PSM) real-world study in the Chinese population. We also analyzed subgroups of patients who may respond differently to the two treatment regimens, which can help clinicians make better treatment options. Furthermore, the independent prognostic factors were identified based on the univariate and multivariate analyses using the Cox proportional hazards model, and a nomogram prognostic model that integrated both the treatment regimens and clinicopathological factors was established.

## Materials and methods

### Study design and participants

**Figure 1:**
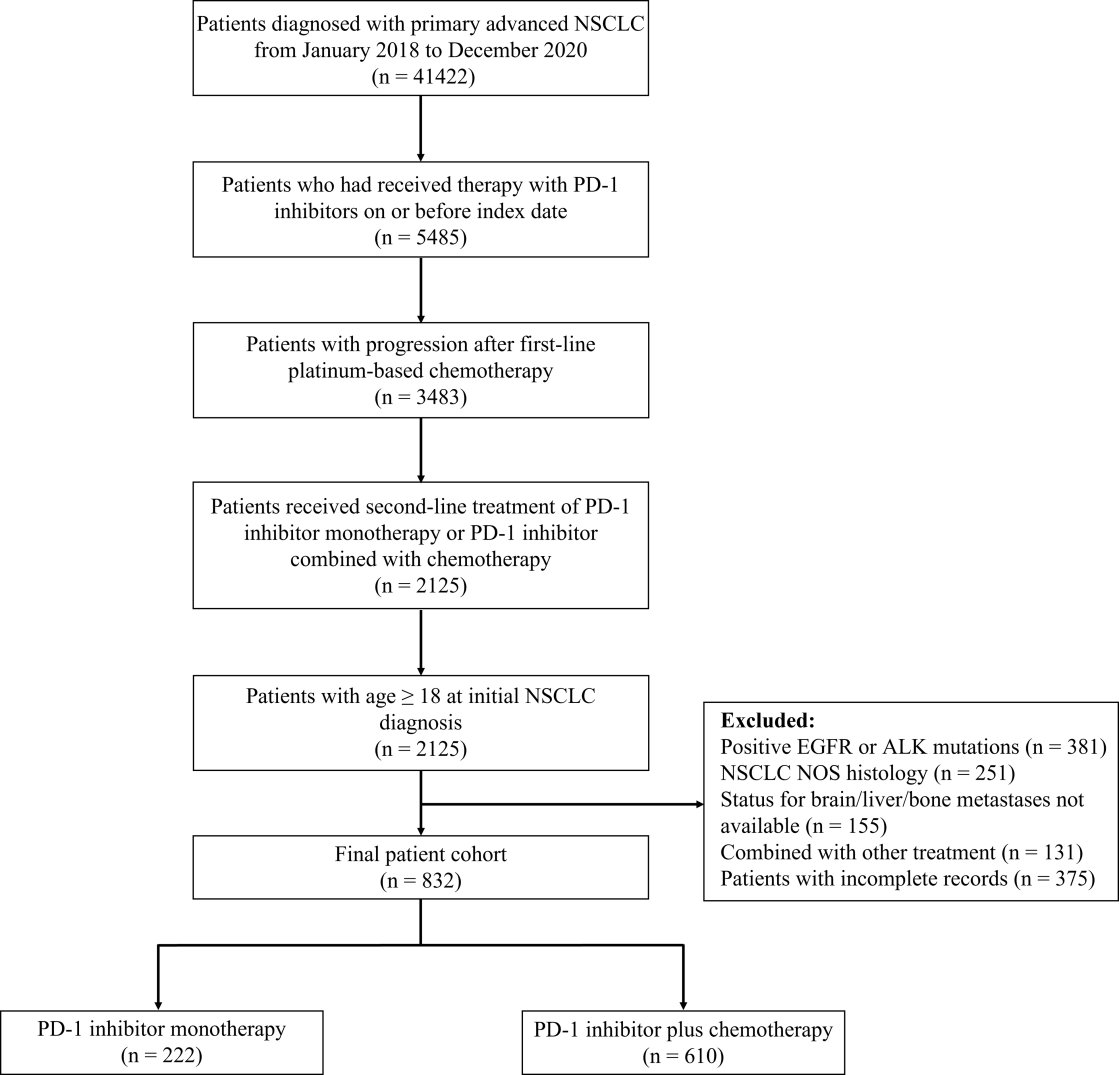
Study flow diagram. NSCLC, non-small-cell lung cancer; PD-1, programmed death 1; EGFR, epidermal growth factor receptor; ALK, anaplastic lymphoma kinase; NOS, not otherwise specified.

This retrospective study evaluated clinical outcomes of real-world patients with confirmed advanced (stage IIIB-IV) NSCLC who received either second-line PD-1 inhibitor monotherapy or PD-1 inhibitor plus chemotherapy after platinum-based chemotherapy. Patient data were extracted from electronic health records in the National Cancer Center database, a longitudinal, demographically and geographically diverse database of approximately 10.0 million patients in China. To protect the privacy of study subjects, deidentified patient data were obtained. The National Cancer Center database contains detailed cancer-related information including basic clinical characteristics, PD-L1 testing information, status for distant metastasis, treatment regimen, and survival time of patients. In this study, patients were excluded if they had positive EGFR or ALK mutations, NSCLC NOS histology, status for distant metastasis not available, combined with other drugs, and incomplete records. The study flow diagram is detailed in **Figure 1**.

This study was approved by the Ethics Committee of Chinese National Cancer Center Hospital, Chinese Academy of Medical Sciences and Peking Union Medical College (No: 20/062-2258), and was in accordance with the ethical guidelines of the Declaration of Helsinki. Written informed consent was waived because the database contained only deidentified data to protect patient confidentiality.

### Study objectives

As of the data cutoff on May 30, 2021, the median follow-up time was 9.2 months. The primary and secondary objectives used for comparison were OS and time to treatment discontinuation (TTD), respectively. OS was defined as the time from the start of second-line PD-1 inhibitor monotherapy or PD-1 inhibitor plus chemotherapy to death or last follow-up. TTD was defined as the time from the start of second-line PD-1 inhibitor monotherapy or PD-1 inhibitor plus chemotherapy to treatment discontinuation for any reason, including disease progression, treatment toxicity, or death. Patients who were alive at the end of study period were censored at the date of last follow-up.

### Statistical analysis

Descriptive statistics were used to describe baseline characteristics of all included participants. Chi-square tests were used to examine differences in categorical covariates between the two treatment groups. To establish that these findings were not confounded by differences in baseline characteristics, we performed a propensity score matching analysis to balance between-group differences. Specifically, propensity score matching was performed using a 1:1 nearest-neighbor matching scheme with a caliper size of 0.2, and the patients in the two treatment groups were matched for age, gender, histology, TNM stage, ECOG PS, smoking status, brain metastasis, liver metastasis, and bone metastasis. Survival analysis was performed by applying the Kaplan-Meier method and the log-rank tests. Univariate and multivariate analyses for OS were conducted with Cox proportional hazards regression models to identify independent prognostic factors. The results were presented as hazard ratio and 95% confidence interval. The R package “forestplot” was used to draw forest plots, and the R package “rma” was used to build the nomogram model for predicting the OS. All statistical analyses were performed using the statistical software R (version 4.1.0) and SPSS (version 25). *p* values < 0.05 were considered significant.

## Results

### Patient characteristics

Among 832 eligible advanced NSCLC patients treated with PD-1 inhibitors after first-line platinum-based chemotherapy, 222 (26.7%) received PD-1 inhibitor monotherapy (ICI-mono group), and 610 (73.3%) received PD-1 inhibitor plus chemotherapy (ICI-chemo group). Baseline demographics and clinical characteristics for patients included in the study were presented in **Table 1**. The median age was 61.0 years (range, 27-83 years), and most patients had adenocarcinoma histology (59.0%) and stage IV disease at initial diagnosis (81.0%). There were no significant differences in brain, liver, and bone metastases between the two treatment groups. The most frequently administered PD-1 inhibitor was camrelizumab in 261 (31.4%) of patients, followed by sintilimab (n = 251, 30.2%), pembrolizumab (n = 171, 20.6%), nivolumab (n = 90, 10.8%), and toripalimab (n = 59, 7.1%). Of the 610 patients who received PD-1 inhibitor plus chemotherapy, paclitaxel (n = 212, 34.8%) was the most commonly used chemotherapy drug, followed by gemcitabine (n = 176, 28.9%) and docetaxel (n = 130, 21.3%). Overall, use of ICI monotherapy was significantly more common in patients with age greater than or equal to 65 years, squamous histology, ECOG PS 0-1, and former smoker, while use of ICI plus chemotherapy was more frequently in patients with age younger than 65 years, adenocarcinoma histology, and current smoker.

**Table 1:** Baseline characteristics of the initial cohort and the matched cohort.

Characteristics	Before propensity score matching			After propensity score matching		
	ICI-mono	ICI-chemo	P value	ICI-mono	ICI-chemo	P value
Number of patients	222	610		222	222	
Age			<0.001			0.847
<65	129	435		129	131	
≥65	93	175		93	91	
Gender			0.460			0.495
Male	175	466		175	169	
Female	47	144		47	53	
Histology			0.017			0.634
Adenocarcinoma	116	375		116	121	
Squamous	106	235		106	101	
Disease stage			0.077			0.820
IIIB/IIIC	51	107		51	49	
IV	171	503		171	173	
ECOG PS			<0.001			0.859
0-1	102	170		102	104	
≥2	8	23		8	6	
Unknown	112	417		112	112	
Smoking status			0.024			0.141
Current smoker	13	67		13	22	
Former smoker	91	193		91	76	
Never smoker	64	179		64	57	
Unknown	54	171		54	67	
PD-L1 expression			0.236			0.216
<50%	19	63		19	23	
≥50%	20	36		20	11	
Unknown	183	511		183	188	
EGFR mutation status			0.507			0.905
Negative	43	106		43	44	
Unknown	179	504		179	178	
ALK mutation status			0.938			0.440
Negative	33	92		33	39	
Unknown	189	518		189	183	
Brain metastases			0.838			1.000
Yes	61	172		61	61	
No	161	438		161	161	
Liver metastases			0.288			0.614
Yes	71	172		71	76	
No	151	438		151	146	
Bone metastases			0.078			1.000
Yes	131	318		131	131	
No	91	292		91	91	

ECOG PS, Eastern Cooperative Oncology Group performance status; PD-L1, programmed death ligand 1; EGFR, epidermal growth factor receptor; ALK, anaplastic lymphoma kinase.

Considering the group imbalances in terms of age, histology, smoking status, and ECOG PS in the initial cohort (**Table 1**), a propensity score matching analysis was performed, and the patients in the two treatment groups were matched for age, gender, histology, TNM stage, ECOG PS, smoking status, brain metastasis, liver metastasis, and bone metastasis. After propensity score matching, 444 patients, including 222 patients in the ICI-mono group and 222 in the ICI-chemo group, were enrolled in the study. No significant differences were observed between the two matched groups in terms of baseline characteristics.

### Propensity score-matched analysis for OS and TTD

**Figure 2:**
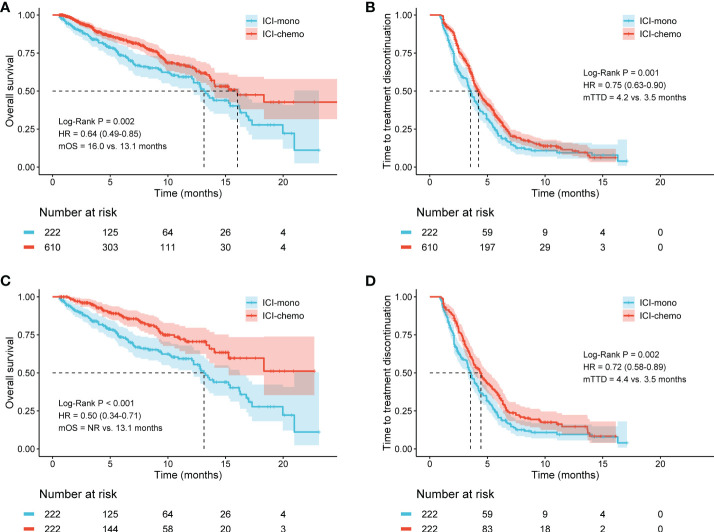
Kaplan-Meier curves for overall survival (OS) and time to treatment discontinuation (TTD) in the initial cohort and the matched cohort. **(A)** ICI-chemo versus ICI-mono for OS in the initial cohort; **(B)** ICI-chemo versus ICI-mono for TTD in the initial cohort; **(C)** ICI-chemo versus ICI-mono for OS in the matched cohort; **(D)** ICI-chemo versus ICI-mono for TTD in the matched cohort. HR, hazard ratio; NR, not reached.

The median follow-up was 9.5 months and 9.1 months in patients treated with ICI monotherapy and ICI plus chemotherapy, respectively. In the initial cohort, patients with advanced NSCLC who received ICI plus chemotherapy had significantly longer OS (HR: 0.64, 95% CI: 0.49-0.85, P = 0.002), with a median OS of 16.0 months compared with 13.1 months for patients who received ICI monotherapy (**Figure 2A**). Similarly, the median TTD was also longer in the ICI-chemo group than that of the ICI-mono group (4.2 months vs. 3.5 months, HR: 0.75, 95% CI: 0.63-0.90, P = 0.001, **Figure 2B**).

To reduce the impact of differences in baseline characteristics among patients treated with ICI monotherapy or ICI plus chemotherapy, we performed a propensity score matching to balance inter-group differences. The results showed that these findings remained significant after propensity score matching. Patients who received ICI plus chemotherapy had significantly longer OS than those who received ICI monotherapy after propensity score matching (NR vs. 13.1 months, HR: 0.50, 95% CI: 0.34-0.71, P < 0.001, **Figure 2C**). Meanwhile, the median TTD was 4.4 months in the ICI-chemo group and 3.5 months in the ICI-mono group (HR: 0.72, 95% CI: 0.58-0.89, P = 0.002, **Figure 2D**).

### Subgroup analysis of survival stratified by treatment group

Subsequently, we performed a subgroup analysis to determine whether patient subgroups can also benefit from the use of ICIs in combination with chemotherapy. In the subgroup of patients younger than 65 years, there was no statistically significant difference in OS between the two treatment groups (15.3 months vs. 15.3 months, P = 0.058, **Figure S1A**), whereas in the subgroup of patients greater than or equal to 65 years, the OS was significantly prolonged in the ICI-chemo group (18.3 months vs. 12.8 months, P = 0.009, **Figure S1B**). Patients who were treated with ICI plus chemotherapy were associated with longer OS for both the lung adenocarcinoma (15.3 months vs. 12.8 months, P = 0.029, **Figure S1C**) and lung squamous carcinoma (16.0 months vs. 13.6 months, P = 0.013, **Figure S1D**) subgroups compared with ICI monotherapy. In addition, we also examined differences in OS between the two treatment groups based on gender (male: P = 0.003; female: P = 0.375), and disease stage (stage IIIB/IIIC: P = 0.026; stage IV: P = 0.010, **Figure S2**).

**Figure 3:**
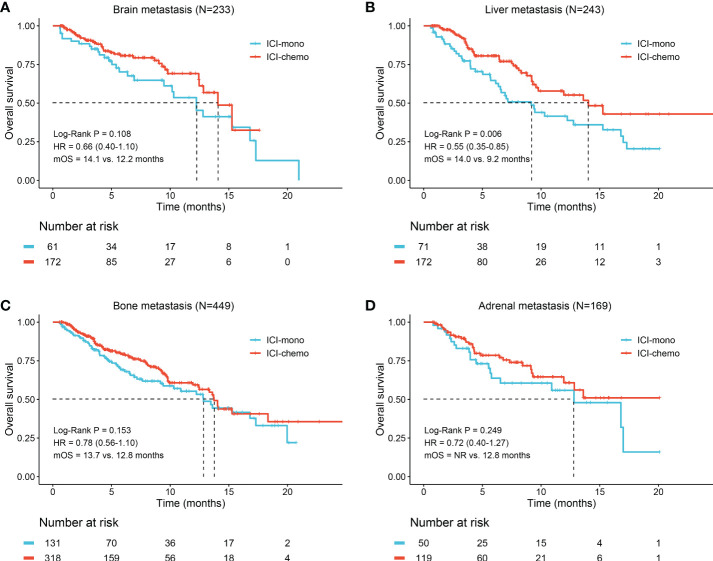
Kaplan-Meier curves of overall survival for patients with different metastatic sites. **(A)** Brain metastasis; **(B)** Liver metastasis; **(C)** Bone metastasis; **(D)** Adrenal metastasis. OS, overall survival; HR, hazard ratio; NR, not reached.

We subsequently analyzed whether patients with brain, liver, bone, and adrenal metastasis could benefit from ICI plus chemotherapy. Survival analysis of unadjusted cohort suggested that patients with liver metastasis (14.0 months vs. 9.2 months, HR: 0.55, 95% CI: 0.35-0.85, P = 0.006, **Figure 3B**) had longer OS when treated with ICI plus chemotherapy. However, there were no significant differences between the two treatment groups in patients with brain metastasis (P = 0.108, **Figure 3A**), bone metastasis (P = 0.153, **Figure 3C**), and adrenal metastasis (P = 0.249, **Figure 3D**). Meanwhile, the median TTD was significantly longer in the ICI-chemo group for patients with brain (4.1 months vs. 3.5 months, P = 0.004), bone (4.2 months vs. 3.6 months, P = 0.035), and adrenal metastasis (4.2 months vs. 3.8 months, P = 0.016, **Figure S3**).

**Figure 4:**
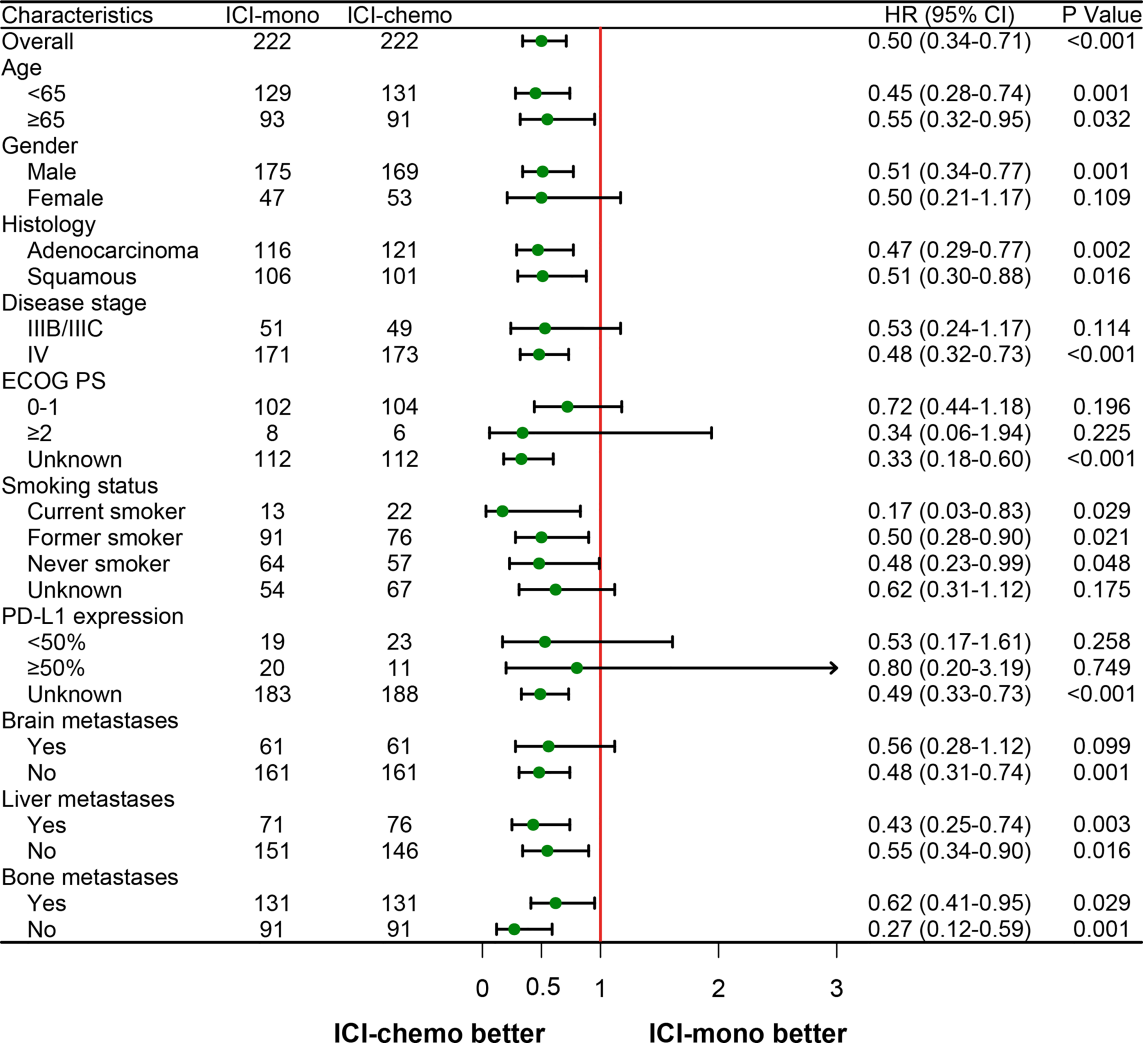
Forest plot of hazard ratios for overall survival by prespecified subgroups after propensity score matching. HR, hazard ratio; ECOG PS, Eastern Cooperative Oncology Group performance status; PD-L1, programmed death ligand 1.

Lastly, to identify subgroups that responded differently to the two treatment options, we performed a subgroup analysis using the initial cohort and the matched cohort. As shown in **Figure S4** and **Figure 4**, ICI-chemo group tended to be associated with longer OS in subgroups of patients with older age, male, adenocarcinoma, squamous carcinoma, stage IV, current smoker, former smoker, no brain metastasis, liver metastasis, and no bone metastasis for both the initial and matched cohorts.

### Univariate and multivariate analyses of overall survival

The results of the univariate and multivariate proportional hazards regression analyses of factors associated with OS in the initial cohort and the matched cohort are shown in **Table S1** and **Table 2**, respectively. Before propensity score matching, the univariate analysis showed that liver metastasis (P = 0.003), bone metastasis (P = 0.002), and treatment regimen (P = 0.002) were significantly associated with OS (**Table S2**). Subsequently, multivariate analysis was performed to identify independent prognostic factors, and the result showed that liver metastasis (HR: 0.747, 95% CI: 0.559-0.998, P = 0.049), bone metastasis (HR: 0.664, 95% CI: 0.487-0.906, P = 0.010), and treatment regimen (HR: 0.674, 95% CI: 0.505-0.900, P = 0.007) were independently associated with OS. Similarly, the univariate and multivariate analyses were also performed in patients after propensity score matching. The results showed that liver metastasis (HR: 0.647, 95% CI: 0.446-0.939, P = 0.022), and treatment regimen (HR: 0.488, 95% CI: 0.337-0.707, P < 0.001) were independent prognostic factors for OS (**Table 2**).

**Table 2:** Univariable and multivariable analyses of overall survival in patients after propensity score matching.

Characteristics	Total (N)	Univariate analysis		Multivariate analysis	
		HR (95% CI)	P value	HR (95% CI)	P value
Age					
<65	260	Reference		Reference	
≥65	184	1.446 (1.014-2.060)	0.041	1.453 (0.998-2.117)	0.051
Gender					
Male	344	Reference		Reference	
Female	100	0.704 (0.448-1.105)	0.127	0.884 (0.504-1.550)	0.667
Histology					
Adenocarcinoma	237	Reference		Reference	
Squamous	207	1.005 (0.712-1.416)	0.979	1.021 (0.697-1.495)	0.915
Disease stage					
IIIB/IIIC	100	Reference		Reference	
IV	344	1.094 (0.716-1.671)	0.679	0.848 (0.533-1.349)	0.486
ECOG PS			0.121		0.218
0-1	206	Reference		Reference	
≥2	14	1.667 (0.764-3.638)	0.199	1.374 (0.590-3.199)	0.461
Unknown	224	0.790 (0.555-1.126)	0.193	0.756 (0.511-1.118)	0.161
Smoking status			0.860		0.604
Current smoker	35	Reference		Reference	
Former smoker	167	0.946 (0.465-1.923)	0.878	0.882 (0.424-1.834)	0.736
Never smoker	121	0.794 (0.379-1.664)	0.542	0.693 (0.310-1.553)	0.373
Unknown	121	0.907 (0.435-1.893)	0.796	1.021 (0.471-2.215)	0.958
PD-L1 expression			0.954		0.967
<50%	42	Reference		Reference	
≥50%	31	1.082 (0.482-2.428)	0.849	0.912 (0.397-2.099)	0.829
Unknown	371	0.982 (0.550-1.752)	0.951	0.992 (0.547-1.797)	0.978
Brain metastases					
Yes	122	Reference		Reference	
No	326	0.739 (0.509-1.074)	0.113	0.765 (0.511-1.144)	0.192
Liver metastases					
Yes	147	Reference		Reference	
No	297	0.636 (0.449-0.900)	0.011	0.647 (0.446-0.939)	0.022
Bone metastases					
Yes	262	Reference		Reference	
No	182	0.699 (0.482-1.013)	0.058	0.681 (0.453-1.025)	0.066
Therapy					
ICI-mono	222	Reference		Reference	
ICI-chemo	222	0.495 (0.344-0.712)	<0.001	0.488 (0.337-0.707)	<0.001

ECOG PS, Eastern Cooperative Oncology Group performance status; PD-L1, programmed death ligand 1; HR, hazard ratio.

In summary, the results presented above indicated that ICI plus chemotherapy was an independent prognostic factor for patients with progression of disease or suboptimal tumor response after first-line platinum-based chemotherapy, and a significant long-term survival benefit was consistently observed when comparing patients treated with second-line ICI monotherapy.

### Development of the prognostic nomogram

**Figure 5:**
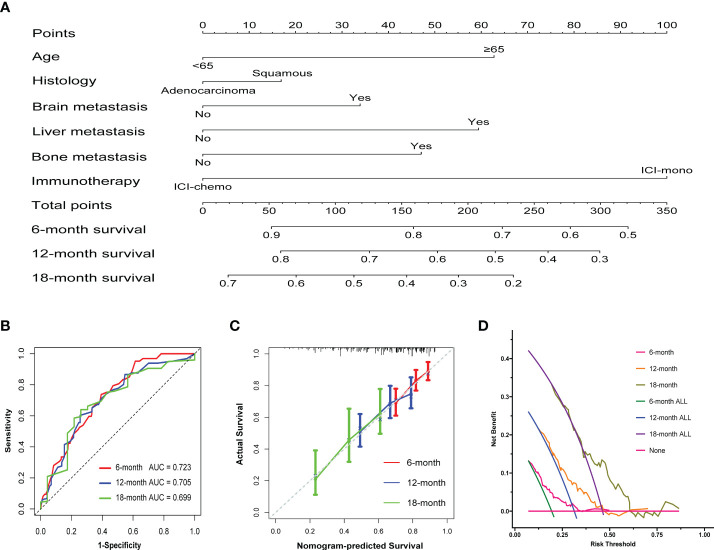
Construction of the nomogram system in patients after propensity score matching. **(A)** Nomogram predicting 6-, 12-, and 18-month overall survival for patients in the matched cohort. A total point was calculated by adding up the scores achieved for each factor. **(B)** ROC curves of the nomogram. **(C)** Calibration curves of the nomogram between predicted and observed 6-, 12-, and 18-month overall survival. The dashed line of 45° represents the perfect prediction of the nomogram. **(D)** Decision curve analysis for the nomogram. The red line at the bottom of the picture represents the assumption that no patients died at 6, 12 or 18 months.

To create a quantitative prognostic model, a nomogram that integrated both the treatment regimens and clinicopathological factors was established by using patients after propensity score matching. According to the nomogram illustrated in this study, a point scale score was assigned to each factor level and the factor scores were summed to obtain a total point for an individual, which can help to predict the 6-, 12- and 18-month overall survival for each patient with advanced NSCLC (**Figure 5A**). As shown, treatment regimens were found to contribute the most to the nomogram model when compared with other clinicopathological factors. We then assessed the predictive accuracy of the nomogram using area under the ROC curve, and the results showed that AUC at 6, 12, and 18 months were 0.723, 0.705, and 0.699, respectively (**Figure 5B**). It appeared that the nomogram calibration curves demonstrated good agreement between prediction and observation in the matched cohort (**Figure 5C**). Moreover, decision curve analyses demonstrated that the nomogram was clinically useful (**Figure 5D**).

## Discussion

As cancer diagnostic and therapeutic technologies continue to advance, the survival time for patients with malignant tumors have been significantly extended (27–30). In recent years, PD-1/PD-L1 inhibitors have changed the treatment paradigm of advanced NSCLC patients without driver mutations (31, 32). However, the optimal timing, dosage and combination of ICI administration remain major clinical problems to clinicians (33–35). Currently, platinum-based combination chemotherapy remains the standard first-line therapy for patients with advanced NSCLC without targetable mutations. ICIs have been approved for advanced NSCLC after disease progression on platinum-based chemotherapy. Indeed, evidence is rapidly accumulating to support the clinical value of combining appropriately dosed chemotherapies with ICIs. Therefore, we designed this retrospective study to evaluate the clinical efficacy of PD-1 inhibitor monotherapy or PD-1 inhibitor plus chemotherapy after first-line platinum-based chemotherapy for patients with advanced NSCLC.

In this study, a total of 832 eligible patients, including 222 received PD-1 inhibitor monotherapy, and 610 received PD-1 inhibitor plus chemotherapy, were enrolled in the analyses. After 1:1 propensity score matching, 222 pairs of patients were successfully matched, and all baseline characteristics were well-balanced. The results showed that patients who received ICI plus chemotherapy had significantly longer OS than those who received ICI monotherapy. CheckMate 017 and CheckMate 057 studies explored the efficacy and safety of nivolumab in second-line or further-line treatments in advanced NSCLC, and the results showed that nivolumab could reduce the risk of death compared with chemotherapy. Similarly, KEYNOTE-189 and KEYNOTE-407 also demonstrated that the addition of pembrolizumab to standard platinum-based chemotherapy was superior to chemotherapy, in terms of PFS and OS. In our study, the median OS of patients in the ICI-chemo and ICI-mono groups was 16.0 and 13.1 months, respectively. Chen et al. analyzed immunotherapy as second‐line treatment and beyond for NSCLC patients in China. The median PFS and OS were 5.0 months and 17.9 months, respectively (36). Simeone et al. enrolled 3290 patients with metastatic NSCLC after first-line chemotherapy, and the median OS was significantly longer in patients who were treated with second-line immunotherapy compared with chemotherapy (17.5months vs. 14.2 months, P < 0.05) (37). Overall, our results were similar to those of previous studies.

Subsequently, we performed multiple subgroup analyses to identify the appropriate subgroups that would benefit from the use of ICIs in combination with chemotherapy. The results indicated that ICI plus chemotherapy was significantly associated with longer OS in patients with older age, male, adenocarcinoma, squamous carcinoma, stage IV, current smoker, and former smoker for both the initial and matched cohorts. Previous studies have shown that patients with distant metastases have a less favorable prognosis (38–40). Landi et al. found that bone metastasis was independently associated with higher risk of death in advanced NSCLC patients treated with nivolumab (41). Powell et al. retrospectively evaluated outcomes in patients with advanced NSCLC to determine whether baseline brain metastases influenced the efficacy of first-line pembrolizumab plus chemotherapy versus chemotherapy alone (42). The results showed that the median OS was 18.8 months with pembrolizumab plus chemotherapy and 7.6 months with chemotherapy in patients with brain metastases. These studies indicated that ICI might improve the survival of patients with metastatic NSCLC. The results of the survival analysis of the present study suggested that patients with liver metastasis (mOS: 14.0 months vs. 9.2 months) and pericardial metastasis (mOS: 11.9 months vs. 3.3 months) had longer OS when treated with ICI plus chemotherapy. While the median OS of patients with brain, bone, and adrenal metastasis in the ICI-chemo group was longer than those in the ICI-mono group, the difference was not statistically significant.

To further explore the role of ICIs among patients with advanced NSCLC after first-line platinum-based chemotherapy, multivariate analyses were performed using Cox proportional hazards regression models. Results of these analyses showed that liver metastasis and treatment regimen were independent prognostic factors for OS in both the initial and matched cohorts. Indeed, previous studies have shown that chemotherapy may increase the sensitivity of cancer cells to ICIs and thus can lead to a synergistic effect between the two (43, 44). These results provide a rationale for combining ICIs and chemotherapy for patients with advanced NSCLC. In general, the therapeutic effect of ICIs plus chemotherapy is superior to that of ICI monotherapy, which suggests that ICIs plus chemotherapy may be a better strategy for patients with advanced NSCLC after first-line platinum-based chemotherapy.

It is well known that nomograms proposed as prognostic tools that can assist in predicting the prognosis for each patient (45). To create a quantitative model for survival prediction, a nomogram was developed for patients with advanced NSCLC. According to the nomogram illustrated in this study, the prognostic score was based on the total points for individuals, which can help to predict the overall survival. Our nomogram may thus represent a useful tool, which can facilitate the clinicians an easy way to predict the prognosis of patients after first-line platinum-based chemotherapy.

To the best of our knowledge, this is the first study to evaluate the clinical response to PD-1 inhibitor monotherapy or PD-1 inhibitor plus chemotherapy after first-line platinum-based chemotherapy for patients with advanced NSCLC in the Chinese population. Our results suggest that patients who received PD-1 inhibitor plus chemotherapy had significantly longer OS than those who received PD-1 inhibitor monotherapy. In addition, we also performed multiple subgroup analyses, and a nomogram was then created to facilitate prediction of survival probability. As real-world data can provide instructive information for clinical practice, the results of this study can provide a valuable reference for patients with advanced NSCLC.

There were also some limitations in the present study. First, as this is a retrospective observational study mainly based on cancer database, we are limited as to how much information can be obtained. For instance, only less than half of included patients have clear ECOG PS, PD-L1 expression, and gene mutation status, which may affect the credibility of subgroup analyses. Second, we cannot be sure whether patients ever received radiotherapy during the treatment period, which may affect patient survival. Third, although all patients enrolled in this study received ICIs after first-line platinum-based chemotherapy, different PD-1 inhibitors and chemotherapy drugs may also have an impact on survival prognosis. More patients and clinicopathological features from prospective multicenter trials are needed to further confirm this prognostic model. Despite the above limitations, our study demonstrated that ICI plus chemotherapy might improve long-term survival in patients with advanced NSCLC after first-line platinum-based chemotherapy compared with ICI monotherapy.

In summary, this study suggests that patients with advanced NSCLC who received ICI plus chemotherapy after first-line platinum-based chemotherapy tended to have longer OS than those who received ICI monotherapy. The multivariate analysis indicated that treatment regimen was an independent prognostic factor for OS. Moreover, a nomogram was created to facilitate the clinicians an easy way to predict the prognosis of patients. We hope these findings can provide a reference for the use of ICIs in patients with advanced NSCLC after platinum-based chemotherapy. Additional prospective studies are needed to confirm the results.

## Data availability statement

Data are available from the corresponding author upon reasonable request.

## Ethics statement

This study was approved by the Ethics Committee of Chinese National Cancer Center Hospital, Chinese Academy of Medical Sciences and Peking Union Medical College (No: 20/062-2258), and was in accordance with the ethical guidelines of the Declaration of Helsinki. Written informed consent was waived because the database contained only deidentified data to protect patient confidentiality.

## Author contributions

Conceptualization: LQ, SG, SD, SJ, XZ. Methodology: LQ, SG, SJ, XZ. Data curation: LQ, SD, SS, YL. Formal analysis: LQ, SD, ZS, TL. Validation: GJ, KL, XS. Writing: LQ, SD, XZ. All authors contributed to the article and approved the submitted version.

## Acknowledgments

We are grateful to all the patients included in this study. The authors also thank Beijing Yiyong Technology Ltd. for statistical assistance.

## Conflict of interest

The authors declare that the research was conducted in the absence of any commercial or financial relationships that could be construed as a potential conflict of interest.

## Publisher’s note

All claims expressed in this article are solely those of the authors and do not necessarily represent those of their affiliated organizations, or those of the publisher, the editors and the reviewers. Any product that may be evaluated in this article, or claim that may be made by its manufacturer, is not guaranteed or endorsed by the publisher.

## References

[1] Sung H Ferlay J Siegel RL Laversanne M Soerjomataram I Jemal A. Global cancer statistics 2020: Globocan estimates of incidence and mortality worldwide for 36 cancers in 185 countries. CA Cancer J Clin (2021) 71(3):209–49. doi: 10.3322/caac.21660 33538338

[2] Barta JA Powell CA Wisnivesky JP. Global epidemiology of lung cancer. Ann Glob Health (2019) 85(1):8. doi: 10.5334/aogh.2419 30741509 PMC6724220

[3] Schabath MB Cote ML. Cancer progress and priorities: Lung cancer. Cancer Epidemiol Biomarkers Prev (2019) 28(10):1563–79. doi: 10.1158/1055-9965.Epi-19-0221 PMC6777859 31575553

[4] Cheng TY Cramb SM Baade PD Youlden DR Nwogu C Reid ME. The international epidemiology of lung cancer: Latest trends, disparities, and tumor characteristics. J Thorac Oncol (2016) 11(10):1653–71. doi: 10.1016/j.jtho.2016.05.021 PMC5512876 27364315

[5] Hu W Bi ZY Chen ZL Liu C Li LL Zhang F. Emerging landscape of circular rnas in lung cancer. Cancer Lett (2018) 427:18–27. doi: 10.1016/j.canlet.2018.04.006 29653267

[6] Mathew M Enzler T Shu CA Rizvi NA. Combining chemotherapy with pd-1 blockade in nsclc. Pharmacol Ther (2018) 186:130–7. doi: 10.1016/j.pharmthera.2018.01.003 29352857

[7] Osmani L Askin F Gabrielson E Li QK. Current who guidelines and the critical role of immunohistochemical markers in the subclassification of non-small cell lung carcinoma (Nsclc): Moving from targeted therapy to immunotherapy. Semin Cancer Biol (2018) 52(Pt 1):103–9. doi: 10.1016/j.semcancer.2017.11.019 PMC5970946 29183778

[8] Tandberg DJ Tong BC Ackerson BG Kelsey CR. Surgery versus stereotactic body radiation therapy for stage I non-small cell lung cancer: A comprehensive review. Cancer (2018) 124(4):667–78. doi: 10.1002/cncr.31196 29266226

[9] Camidge DR Doebele RC Kerr KM. Comparing and contrasting predictive biomarkers for immunotherapy and targeted therapy of nsclc. Nat Rev Clin Oncol (2019) 16(6):341–55. doi: 10.1038/s41571-019-0173-9 30718843

[10] Duma N Santana-Davila R Molina JR. Non-small cell lung cancer: Epidemiology, screening, diagnosis, and treatment. Mayo Clin Proc (2019) 94(8):1623–40. doi: 10.1016/j.mayocp.2019.01.013 31378236

[11] Siegel RL Miller KD Fuchs HE Jemal A. Cancer statistics, 2022. CA Cancer J Clin (2022) 72(1):7–33. doi: 10.3322/caac.21708 35020204

[12] Le Chevalier T Scagliotti G Natale R Danson S Rosell R Stahel R. Efficacy of gemcitabine plus platinum chemotherapy compared with other platinum containing regimens in advanced non-Small-Cell lung cancer: A meta-analysis of survival outcomes. Lung Cancer (2005) 47(1):69–80. doi: 10.1016/j.lungcan.2004.10.014 15603856

[13] Grant MJ Herbst RS Goldberg SB. Selecting the optimal immunotherapy regimen in driver-negative metastatic nsclc. Nat Rev Clin Oncol (2021) 18(10):625–44. doi: 10.1038/s41571-021-00520-1 34168333

[14] Proto C Ferrara R Signorelli D Lo Russo G Galli G Imbimbo M. Choosing wisely first line immunotherapy in non-small cell lung cancer (Nsclc): What to add and what to leave out. Cancer Treat Rev (2019) 75:39–51. doi: 10.1016/j.ctrv.2019.03.004 30954906

[15] Yang Y Yu Y Lu S. Effectiveness of pd-1/Pd-L1 inhibitors in the treatment of lung cancer: Brightness and challenge. Sci China Life Sci (2020) 63(10):1499–514. doi: 10.1007/s11427-019-1622-5 32303964

[16] Salmaninejad A Valilou SF Shabgah AG Aslani S Alimardani M Pasdar A. Pd-1/Pd-L1 pathway: Basic biology and role in cancer immunotherapy. J Cell Physiol (2019) 234(10):16824–37. doi: 10.1002/jcp.28358 30784085

[17] Patsoukis N Wang Q Strauss L Boussiotis VA. Revisiting the pd-1 pathway. Sci Adv (2020) 6(38):eabd2712. doi: 10.1126/sciadv.abd2712 32948597 PMC7500922

[18] Paz-Ares L Vicente D Tafreshi A Robinson A Soto Parra H Mazières J. A randomized, placebo-controlled trial of pembrolizumab plus chemotherapy in patients with metastatic squamous nsclc: Protocol-specified final analysis of keynote-407. J Thorac Oncol (2020) 15(10):1657–69. doi: 10.1016/j.jtho.2020.06.015 32599071

[19] Gadgeel S Rodríguez-Abreu D Speranza G Esteban E Felip E Dómine M. Updated analysis from keynote-189: Pembrolizumab or placebo plus pemetrexed and platinum for previously untreated metastatic nonsquamous non-Small-Cell lung cancer. J Clin Oncol (2020) 38(14):1505–17. doi: 10.1200/jco.19.03136 32150489

[20] Borghaei H Paz-Ares L Horn L Spigel DR Steins M Ready NE. Nivolumab versus docetaxel in advanced nonsquamous non-Small-Cell lung cancer. N Engl J Med (2015) 373(17):1627–39. doi: 10.1056/NEJMoa1507643 PMC5705936 26412456

[21] Brahmer J Reckamp KL Baas P Crinò L Eberhardt WE Poddubskaya E. Nivolumab versus docetaxel in advanced squamous-cell non-Small-Cell lung cancer. N Engl J Med (2015) 373(2):123–35. doi: 10.1056/NEJMoa1504627 PMC4681400 26028407

[22] Zhou C Chen G Huang Y Zhou J Lin L Feng J. Camrelizumab plus carboplatin and pemetrexed versus chemotherapy alone in chemotherapy-naive patients with advanced non-squamous non-Small-Cell lung cancer (Camel): A randomised, open-label, multicentre, phase 3 trial. Lancet Respir Med (2021) 9(3):305–14. doi: 10.1016/s2213-2600(20)30365-9 33347829

[23] Wang Z Ying J Xu J Yuan P Duan J Bai H. Safety, antitumor activity, and pharmacokinetics of toripalimab, a programmed cell death 1 inhibitor, in patients with advanced non-small cell lung cancer: A phase 1 trial. JAMA Netw Open (2020) 3(10):e2013770. doi: 10.1001/jamanetworkopen.2020.13770 33017026 PMC7536589

[24] Yang Y Wang Z Fang J Yu Q Han B Cang S. Efficacy and safety of sintilimab plus pemetrexed and platinum as first-line treatment for locally advanced or metastatic nonsquamous nsclc: A randomized, double-blind, phase 3 study (Oncology program by innovent anti-Pd-1-11). J Thorac Oncol (2020) 15(10):1636–46. doi: 10.1016/j.jtho.2020.07.014 32781263

[25] Pasello G Pavan A Attili I Bortolami A Bonanno L Menis J. Real world data in the era of immune checkpoint inhibitors (Icis): Increasing evidence and future applications in lung cancer. Cancer Treat Rev (2020) 87:102031. doi: 10.1016/j.ctrv.2020.102031 32446182

[26] Eisenhauer EA. Real-world evidence in the treatment of ovarian cancer. Ann Oncol (2017) 28(suppl_8):viii61–viii5. doi: 10.1093/annonc/mdx443 29232466

[27] Boucherit N Gorvel L Olive D. 3d tumor models and their use for the testing of immunotherapies. Front Immunol (2020) 11:603640. doi: 10.3389/fimmu.2020.603640 33362787 PMC7758240

[28] Liu Y Bhattarai P Dai Z Chen X. Photothermal therapy and photoacoustic imaging *Via* nanotheranostics in fighting cancer. Chem Soc Rev (2019) 48(7):2053–108. doi: 10.1039/c8cs00618k PMC6437026 30259015

[29] Zhang Y Li M Gao X Chen Y Liu T. Nanotechnology in cancer diagnosis: Progress, challenges and opportunities. J Hematol Oncol (2019) 12(1):137. doi: 10.1186/s13045-019-0833-3 31847897 PMC6918551

[30] Herrmann K Schwaiger M Lewis JS Solomon SB McNeil BJ Baumann M. Radiotheranostics: A roadmap for future development. Lancet Oncol (2020) 21(3):e146–e56. doi: 10.1016/s1470-2045(19)30821-6 PMC7367151 32135118

[31] Brozos-Vázquez EM Díaz-Peña R García-González J León-Mateos L Mondelo-Macía P Peña-Chilet M. Immunotherapy in nonsmall-cell lung cancer: Current status and future prospects for liquid biopsy. Cancer Immunol Immunother (2021) 70(5):1177–88. doi: 10.1007/s00262-020-02752-z PMC10991125 33113004

[32] Eguren-Santamaria I Sanmamed MF Goldberg SB Kluger HM Idoate MA Lu BY. Pd-1/Pd-L1 blockers in nsclc brain metastases: Challenging paradigms and clinical practice. Clin Cancer Res (2020) 26(16):4186–97. doi: 10.1158/1078-0432.Ccr-20-0798 32354698

[33] Barroso-Sousa R Barry WT Garrido-Castro AC Hodi FS Min L Krop IE. Incidence of endocrine dysfunction following the use of different immune checkpoint inhibitor regimens: A systematic review and meta-analysis. JAMA Oncol (2018) 4(2):173–82. doi: 10.1001/jamaoncol.2017.3064 PMC5838579 28973656

[34] Bagchi S Yuan R Engleman EG. Immune checkpoint inhibitors for the treatment of cancer: Clinical impact and mechanisms of response and resistance. Annu Rev Pathol (2021) 16:223–49. doi: 10.1146/annurev-pathol-042020-042741 33197221

[35] Kwon M Jung H Nam GH Kim IS. The right timing, right combination, right sequence, and right delivery for cancer immunotherapy. J Control Release (2021) 331:321–34. doi: 10.1016/j.jconrel.2021.01.009 33434599

[36] Chen M Li Q Xu Y Zhao J Zhang L Wei L. Immunotherapy as second-line treatment and beyond for non-small cell lung cancer in a single center of China: Outcomes, toxicities, and clinical predictive factors from a real-world retrospective analysis. Thorac Cancer (2020) 11(7):1955–62. doi: 10.1111/1759-7714.13488 PMC7327684 32468726

[37] Simeone JC Nordstrom BL Patel K Klein AB. Treatment patterns and overall survival in metastatic non-Small-Cell lung cancer in a real-world, us setting. Future Oncol (2019) 15(30):3491–502. doi: 10.2217/fon-2019-0348 31497994

[38] Bongiovanni A Foca F Menis J Stucci SL Artioli F Guadalupi V. Immune checkpoint inhibitors with or without bone-targeted therapy in nsclc patients with bone metastases and prognostic significance of neutrophil-to-Lymphocyte ratio. Front Immunol (2021) 12:697298. doi: 10.3389/fimmu.2021.697298 34858389 PMC8631508

[39] Vokes EE Ready N Felip E Horn L Burgio MA Antonia SJ. Nivolumab versus docetaxel in previously treated advanced non-Small-Cell lung cancer (Checkmate 017 and checkmate 057): 3-year update and outcomes in patients with liver metastases. Ann Oncol (2018) 29(4):959–65. doi: 10.1093/annonc/mdy041 29408986

[40] Nogami N Barlesi F Socinski MA Reck M Thomas CA Cappuzzo F. Impower150 final exploratory analyses for atezolizumab plus bevacizumab and chemotherapy in key nsclc patient subgroups with egfr mutations or metastases in the liver or brain. J Thorac Oncol (2022) 17(2):309–23. doi: 10.1016/j.jtho.2021.09.014 34626838

[41] Landi L D'Incà F Gelibter A Chiari R Grossi F Delmonte A. Bone metastases and immunotherapy in patients with advanced non-Small-Cell lung cancer. J Immunother Cancer (2019) 7(1):316. doi: 10.1186/s40425-019-0793-8 31752994 PMC6868703

[42] Powell SF Rodríguez-Abreu D Langer CJ Tafreshi A Paz-Ares L Kopp HG. Outcomes with pembrolizumab plus platinum-based chemotherapy for patients with nsclc and stable brain metastases: Pooled analysis of keynote-021, -189, and -407. J Thorac Oncol (2021) 16(11):1883–92. doi: 10.1016/j.jtho.2021.06.020 34265431

[43] Emens LA Middleton G. The interplay of immunotherapy and chemotherapy: Harnessing potential synergies. Cancer Immunol Res (2015) 3(5):436–43. doi: 10.1158/2326-6066.Cir-15-0064 PMC5012642 25941355

[44] Yu WD Sun G Li J Xu J Wang X. Mechanisms and therapeutic potentials of cancer immunotherapy in combination with radiotherapy and/or chemotherapy. Cancer Lett (2019) 452:66–70. doi: 10.1016/j.canlet.2019.02.048 30902563

[45] Iasonos A Schrag D Raj GV Panageas KS. How to build and interpret a nomogram for cancer prognosis. J Clin Oncol (2008) 26(8):1364–70. doi: 10.1200/jco.2007.12.9791 18323559

