# Supplementary material for: Immune checkpoint inhibitors alone or in combination with chemotherapy for treatment of advanced non-small cell lung cancer after first-line platinum-based chemotherapy: A propensity score matching analysis
Source: Front Oncol. 2022 Nov 29;12:974227. doi: 10.3389/fonc.2022.974227 (PMC9745307; doi:10.3389/fonc.2022.974227)
Supplement: Supplementary file 1 [file DataSheet_1.docx]

Supplementary Material

## Supplementary Figures


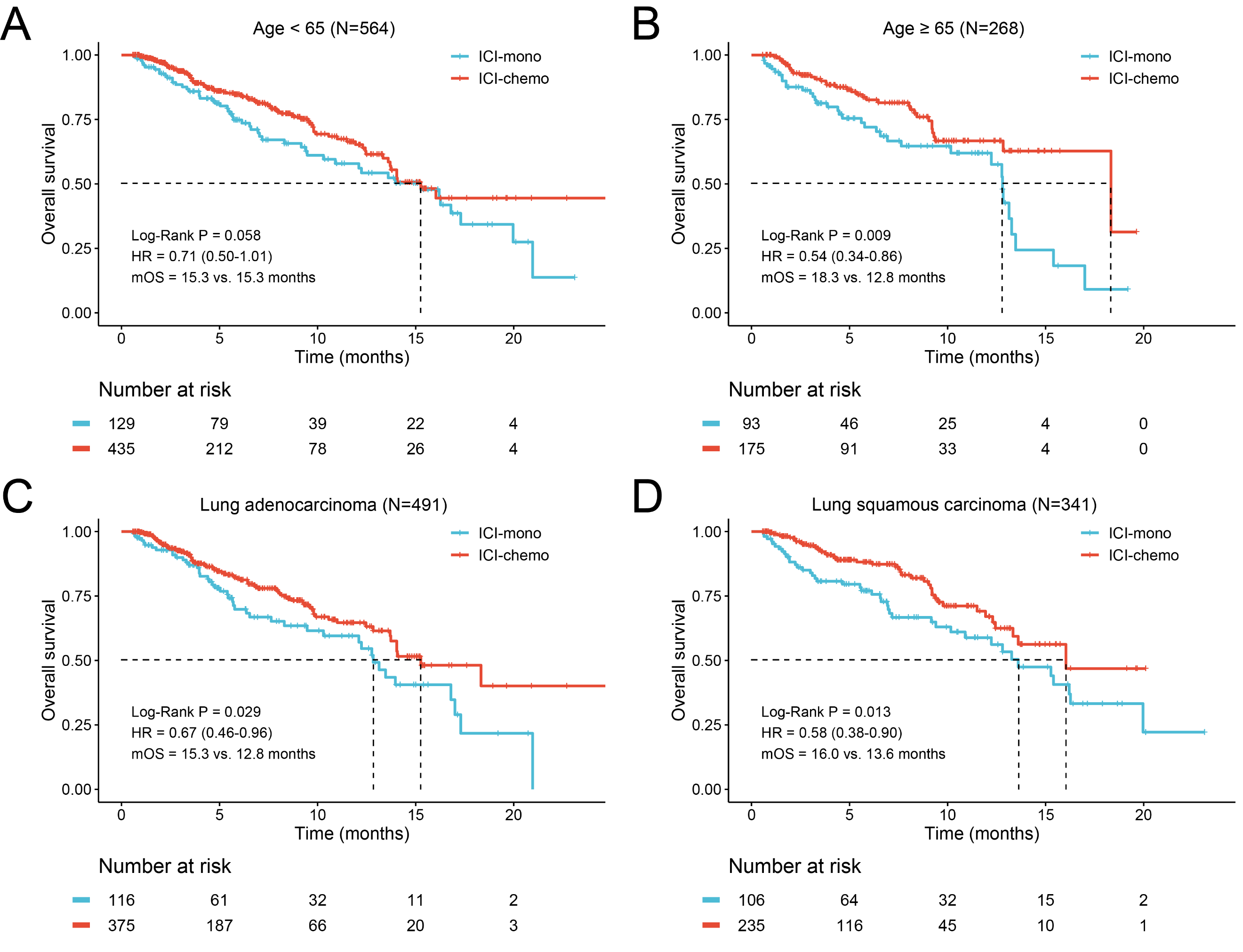


**Supplementary Figure 1.** Kaplan-Meier curves of overall survival for patients before propensity score matching. (A) Age younger than 65 years; (B) Age greater than or equal to 65 years; (C) Lung adenocarcinoma; (D) Lung squamous carcinoma. ICI-mono, immune checkpoint inhibitor monotherapy; ICI-chemo, immune checkpoint inhibitor plus chemotherapy; OS, overall survival; HR, hazard ratio; NR, not reached.


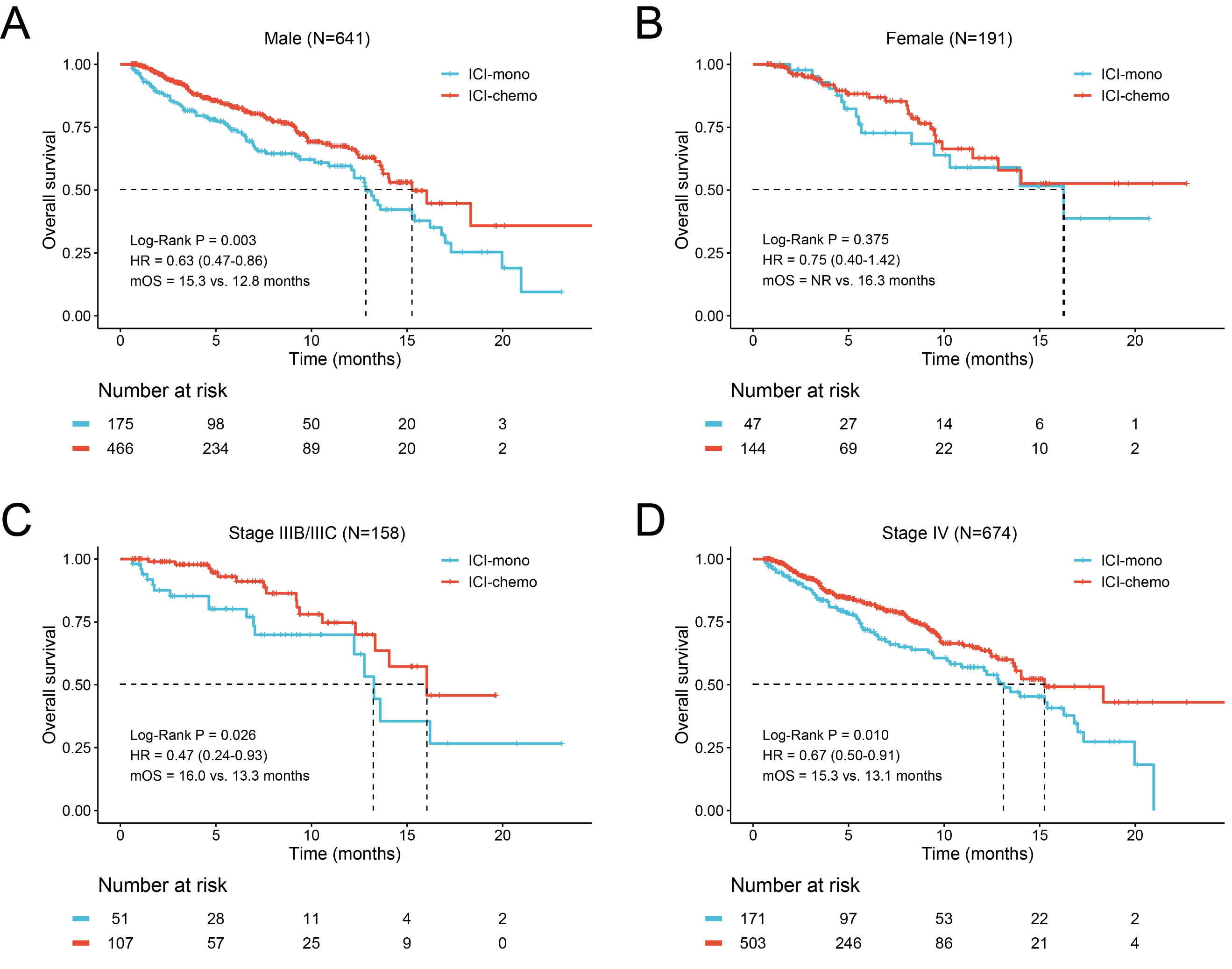


**Supplementary Figure 2.** Kaplan-Meier curves of overall survival for patients before propensity score matching. (A) Male; (B) Female; (C) Stage IIIB/IIIC; (D) Stage IV. ICI-mono, immune checkpoint inhibitor monotherapy; ICI-chemo, immune checkpoint inhibitor plus chemotherapy; OS, overall survival; HR, hazard ratio; NR, not reached.


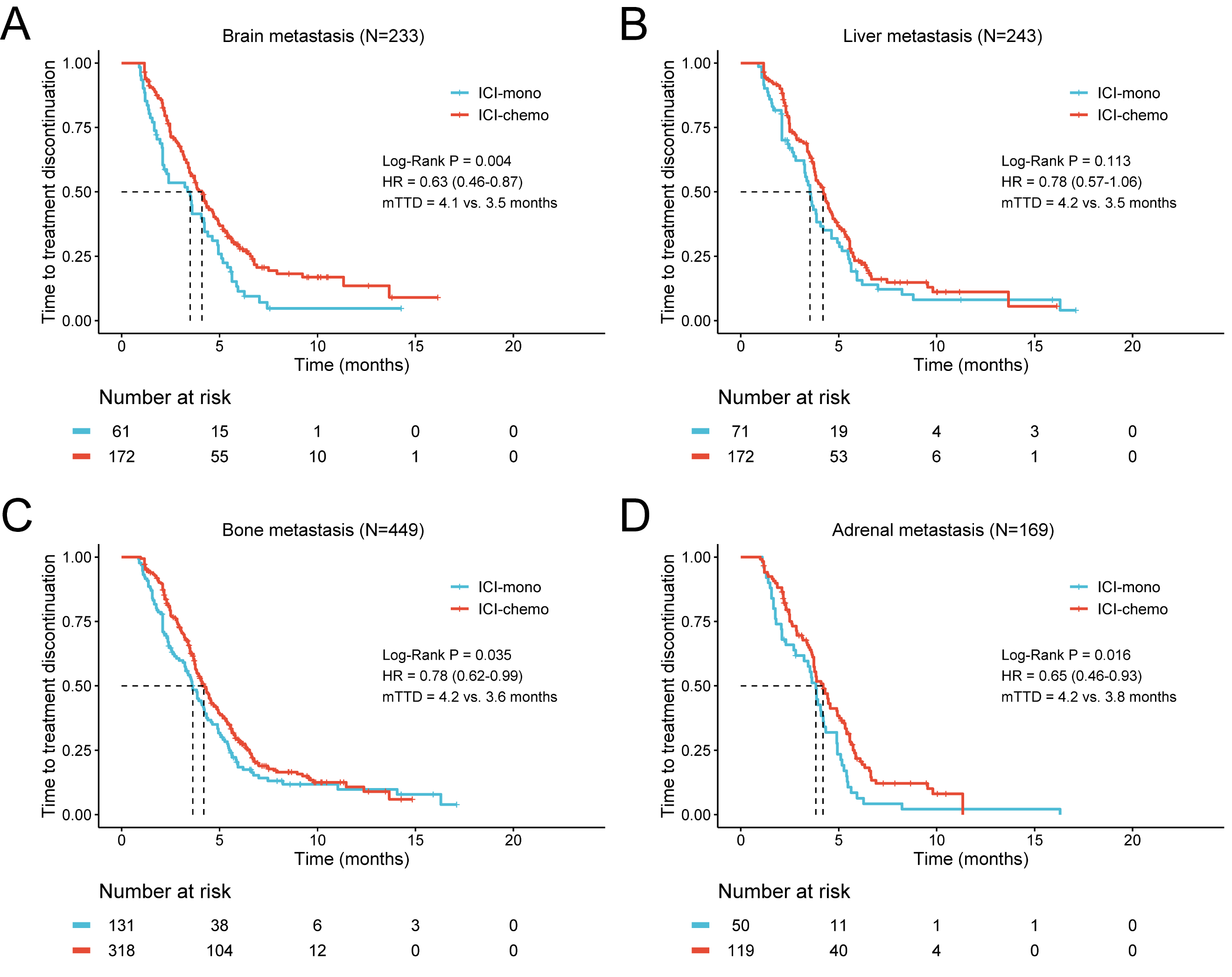


**Supplementary Figure 3.** Kaplan-Meier curves of time to treatment discontinuation for patients with different metastatic sites. (A) Brain metastasis; (B) Liver metastasis; (C) Bone metastasis; (D) Adrenal metastasis. TTD, time to treatment discontinuation; HR, hazard ratio.


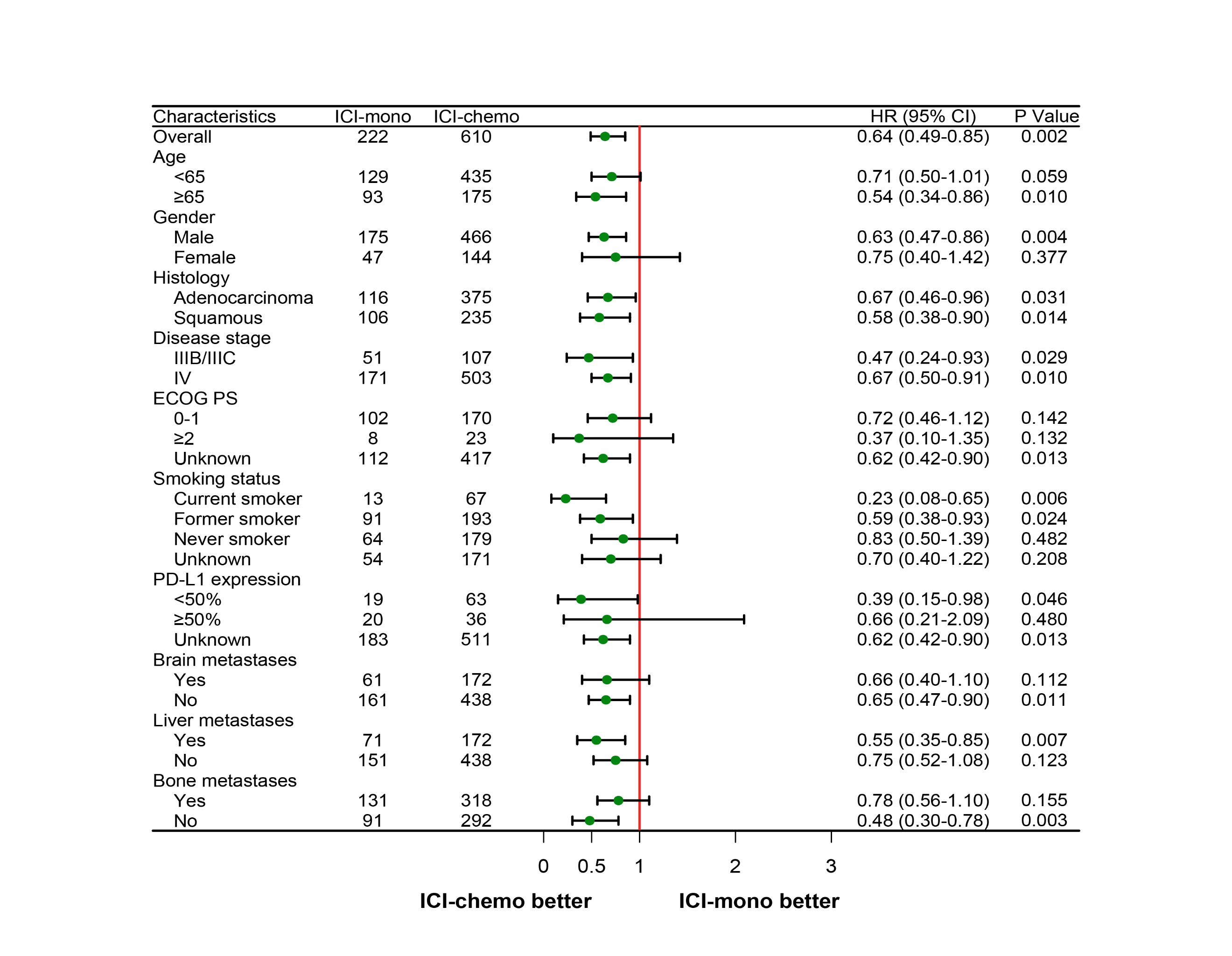


**Supplementary Figure 4.** Forest plot of hazard ratios for overall survival by prespecified subgroups before propensity score matching. ICI-mono, immune checkpoint inhibitor monotherapy; ICI-chemo, immune checkpoint inhibitor plus chemotherapy; HR, hazard ratio; ECOG PS, Eastern Cooperative Oncology Group performance status; PD-L1, programmed death ligand 1.

## Supplementary Tables

**Supplementary Table 1.** Summary of clinical trials of immune checkpoint inhibitors in non-small cell lung cancer.

| Clinical trial | Histology | Design | Immunotherapy | Control |
| --- | --- | --- | --- | --- |
| **First-line** |  |  |  |  |
| KEYNOTE-024 | NSCLC | Pembrolizumab vs. chemotherapy | mOS 30.0 months | mOS 14.2 months |
| KEYNOTE-042 | NSCLC | Pembrolizumab vs. chemotherapy | mOS 16.7 months | mOS 12.1 months |
| KEYNOTE-189 | Nonsquamous | Pembrolizumab plus chemotherapy vs. chemotherapy | mOS 22.0 months | mOS 10.6 months |
| KEYNOTE-407 | Squamous | Pembrolizumab plus chemotherapy vs. chemotherapy | mOS 15.9 months | mOS 11.3 months |
| CheckMate 026 | NSCLC | Nivolumab vs. chemotherapy | mOS 14.4 months | mOS 13.2 months |
| **Second-line or later** | | | | |
| KEYNOTE-010 | NSCLC | Pembrolizumab vs. chemotherapy | mOS 12.7 months | mOS 8.5 months |
| CheckMate 017 | Squamous | Nivolumab vs. chemotherapy | mOS 9.2 months | mOS 6.0 months |
| CheckMate 057 | Nonsquamous | Nivolumab vs. chemotherapy | mOS 12.2 months | mOS 9.5 months |
| Impower 150 | Nonsquamous | Atezolizumab plus chemotherapy vs. chemotherapy | mOS 19.2 months | mOS 14.7 months |

NSCLC, non-small cell lung cancer; OS, overall survival.

**Supplementary Table 2.** Univariable and multivariable analyses of overall survival in patients before propensity score matching.

| Characteristics | Total (N) | Univariate analysis | |  | Multivariate analysis | |
| --- | --- | --- | --- | --- | --- | --- |
|  |  | HR (95% CI) | P value |  | HR (95% CI) | P value |
| Age |  |  |  |  |  |  |
| <65 | 564 | Reference |  |  | Reference |  |
| ≥65 | 268 | 1.191 (0.894-1.588) | 0.232 |  | 1.168 (0.868-1.573) | 0.305 |
| Gender |  |  |  |  |  |  |
| Male | 641 | Reference |  |  | Reference |  |
| Female | 191 | 0.798 (0.567-1.122) | 0.194 |  | 0.740 (0.494-1.109) | 0.145 |
| Histology |  |  |  |  |  |  |
| Adenocarcinoma | 491 | Reference |  |  | Reference |  |
| Squamous | 341 | 0.902 (0.684-1.188) | 0.462 |  | 0.932 (0.693-1.253) | 0.639 |
| Disease stage |  |  |  |  |  |  |
| IIIB/IIIC | 158 | Reference |  |  | Reference |  |
| IV | 674 | 1.338 (0.923-1.940) | 0.125 |  | 1.095 (0.738-1.625) | 0.653 |
| ECOG PS |  |  | 0.603 |  |  | 0.825 |
| 0-1 | 272 | Reference |  |  | Reference |  |
| ≥2 | 31 | 1.236 (0.657-2.324) | 0.511 |  | 1.228 (0.639-2.359) | 0.537 |
| Unknown | 529 | 0.923 (0.695-1.225) | 0.578 |  | 1.016 (0.752-1.372) | 0.918 |
| Smoking status |  |  | 0.911 |  |  | 0.901 |
| Current smoker | 80 | Reference |  |  | Reference |  |
| Former smoker | 284 | 1.189 (0.683-2.069) | 0.541 |  | 1.149 (0.654-2.018) | 0.630 |
| Never smoker | 243 | 1.233 (0.702-2.166) | 0.467 |  | 1.236 (0.682-2.239) | 0.485 |
| Unknown | 225 | 1.171 (0.661-2.077) | 0.589 |  | 1.221 (0.679-2.197) | 0.505 |
| PD-L1 expression |  |  | 0.261 |  |  | 0.159 |
| <50% | 82 | Reference |  |  | Reference |  |
| ≥50% | 56 | 1.078 (0.534-2.175) | 0.834 |  | 0.960 (0.470-1.959) | 0.911 |
| Unknown | 694 | 1.414 (0.868-2.302) | 0.164 |  | 1.427 (0.869-2.343) | 0.159 |
| Brain metastases |  |  |  |  |  |  |
| Yes | 233 | Reference |  |  | Reference |  |
| No | 599 | 0.824 (0.614-1.106) | 0.198 |  | 0.877 (0.646-1.190) | 0.399 |
| Liver metastases |  |  |  |  |  |  |
| Yes | 243 | Reference |  |  | Reference |  |
| No | 589 | 0.657 (0.498-0.868) | 0.003 |  | 0.747 (0.559-0.998) | 0.049 |
| Bone metastases |  |  |  |  |  |  |
| Yes | 449 | Reference |  |  | Reference |  |
| No | 383 | 0.634 (0.476-0.846) | 0.002 |  | 0.664 (0.487-0.906) | 0.010 |
| Therapy |  |  |  |  |  |  |
| ICI-mono | 222 | Reference |  |  | Reference |  |
| ICI-chemo | 610 | 0.644 (0.489-0.850) | 0.002 |  | 0.674 (0.505-0.900) | 0.007 |

ICI-mono, immune checkpoint inhibitor monotherapy; ICI-chemo, immune checkpoint inhibitor plus chemotherapy; ECOG PS, Eastern Cooperative Oncology Group performance status; PD-L1, programmed death ligand 1; HR, hazard ratio.
